# Cardiac and Vascular Adaptation During Pregnancy in Asian and Caucasian Women: Insights from a Prospective Cohort Study

**DOI:** 10.3390/jcm15020756

**Published:** 2026-01-16

**Authors:** Andrea Sonaglioni, Irene Sutti, Giuditta Ferrara, Marta Ruggiero, Giovanna Margola, Gian Luigi Nicolosi, Stefano Bianchi, Michele Lombardo, Massimo Baravelli

**Affiliations:** 1 Division of Cardiology, Istituto di Ricovero e Cura a Carattere Scientifico (IRCCS) MultiMedica, 20123 Milan, Italy; michele.lombardo@multimedica.it (M.L.); massimo.baravelli@multimedica.it (M.B.); 2 Department of Obstetrics and Gynecology, Macedonio Melloni Hospital, 20129 Milan, Italy; irene.sutti@unimi.it; 3 Department of Obstetrics and Gynecology, IRCCS MultiMedica, 20123 Milan, Italy; giuditta.ferrara@multimedica.it (G.F.); marta.ruggiero@multimedica.it (M.R.); giovanna.margola@studenti.unimi.it (G.M.); stefano.bianchi@unimi.it (S.B.); 4 Division of Cardiology, Policlinico San Giorgio, 33170 Pordenone, Italy; gianluigi.nicolosi@gmail.com

**Keywords:** pregnancy, Asian ethnicity, cardiac mechanics, speckle-tracking echocardiography, carotid ultrasonography

## Abstract

**Background/Objectives:** Ethnicity is associated with differences in cardiac structure and function in non-pregnant populations, but pregnancy-specific data—particularly for myocardial deformation—remain limited. We investigated whether ethnicity influences cardiac geometry, biventricular and biatrial mechanics, hemodynamics, and carotid vascular indices in healthy women during the third trimester of pregnancy. **Methods:** In this prospective, monocentric study, 80 healthy women with singleton third-trimester pregnancies were enrolled, including 40 Asian and 40 Caucasian women matched for age and body mass index. All participants underwent standardized clinical and laboratory evaluation, comprehensive transthoracic echocardiography with Doppler, speckle-tracking analysis of both ventricles and atria, and bilateral carotid ultrasonography. Logistic regression analyses were performed in Asian women to identify correlates of supranormal left ventricular ejection fraction (LVEF ≥ 70%) and enhanced left ventricular global longitudinal strain (LV-GLS > 20%). **Results:** Age and gestational age were similar between groups, whereas body surface area was lower in Asian women (1.65 ± 0.12 vs. 1.77 ± 0.15 m^2^, *p* < 0.001). Asian women exhibited smaller left ventricular dimensions and volumes but higher LVEF (median 71.6% vs. 66.4%, *p* < 0.001). Heart rate and blood pressure were comparable, whereas stroke volume [45.5 ± 9.6 vs. 68.0 (48.9–110) mL, *p* < 0.001] and cardiac output (3.9 ± 0.9 vs. 4.9 ± 0.8 L/min, *p* < 0.001) were lower in Asian women, who also demonstrated higher total peripheral resistance and lower ventricular–arterial coupling (0.31 ± 0.09 vs. 0.37 ± 0.07, *p* = 0.001). Speckle-tracking echocardiography revealed higher LV-GLS (21.9 ± 1.9% vs. 20.5 ± 2.0%, *p* = 0.002), higher LV global circumferential strain, enhanced right ventricular longitudinal strain, and higher reservoir strain of both atria in Asian women. Carotid ultrasonography showed smaller common carotid diameter and cross-sectional area in Asian women (10.7 ± 2.5 vs. 13.7 ± 2.3 mm^2^, *p* < 0.001). In regression analyses, supranormal LVEF was independently associated with smaller LV end-diastolic diameter (OR 0.39, 95% CI 0.16–0.97), while enhanced LV-GLS was independently associated with lower neutrophil-to-lymphocyte ratio (OR 0.04, 95% CI 0.00–0.87). **Conclusions:** Ethnicity is associated with multidimensional differences in cardiac geometry, myocardial mechanics, vascular load, and carotid structure in healthy third-trimester pregnancy. Ethnicity-aware interpretation and tailored reference ranges may improve the accuracy of echocardiographic assessment during late gestation.

## 1. Introduction

Cardiovascular disease remains the leading cause of maternal mortality worldwide [1,2]. Nearly two-thirds of maternal deaths related to cardiovascular conditions are considered potentially preventable [3], with cardiac and coronary disease as well as cardio-myopathy accounting for approximately 21% of cases [4]. Pregnancy is characterized by profound hemodynamic changes, including increases in cardiac output, circulating blood volume, and heart rate, which may reveal previously unrecognized cardiovascular abnormalities or worsen pre-existing disease. Consequently, a detailed evaluation of cardiac structure and function in healthy pregnant women is essential to characterize physiological cardiovascular adaptation and to provide an appropriate reference for identifying maladaptive changes, such as those occurring in women with obesity [5] or in pregnancies complicated by gestational hypertension [6] or gestational diabetes mellitus [7].

Transthoracic echocardiography (TTE) is the most widely used non-invasive modality for cardiovascular assessment during pregnancy because of its safety, accessibility, and reproducibility. TTE allows comprehensive evaluation of cardiac chamber dimensions, left ventricular (LV) diastolic function, biventricular systolic performance, valvular function, and pulmonary hemodynamics. Although several investigations over the past decade have attempted to define normal echocardiographic parameters during pregnancy, most have been limited by small sample sizes and heterogeneous timing of examinations across gestation, thereby hindering the establishment of robust pregnancy-specific reference ranges [8,9,10].

Large population-based echocardiographic studies in healthy, non-pregnant individuals without cardiovascular, renal, or metabolic disease have consistently demonstrated important ethnic differences in cardiac structure and function. These differences involve left atrial (LA) and LV dimensions and volumes, left ventricular mass index (LVMi), and left ventricular ejection fraction (LVEF) [11]. Notably, the lower reference limit of LVEF has been reported to be approximately 6% lower in Europeans compared with East Asians and 2% lower compared with South Asians, with statistically significant differences observed in both sexes. In addition, upper reference limits for left ventricular end-diastolic diameter (LVEDD), left ventricular end-systolic diameter (LVESD), LV end-diastolic and end-systolic volumes, and LA antero-posterior (A–P) diameter are higher in European populations than in Asian populations.

Higher LVEF values observed in Asian individuals compared with Europeans have been associated with smaller cardiac chamber dimensions. This relationship suggests that individuals with smaller hearts may rely on greater systolic shortening to maintain adequate cardiac output [12]. Moreover, several studies have shown that LVEF may be overestimated in individuals with normal myocardial function but small ventricular volumes, highlighting the limitations of LVEF as a sole index of systolic performance in certain populations [13,14].

Despite growing awareness of ethnicity-related differences in cardiac structure and function in non-pregnant populations, these differences have been poorly investigated during pregnancy. Wilkie et al. [15] reported higher LVEF values in Asian compared with White pregnant women, while cardiac geometry did not differ significantly between groups and all echocardiographic parameters remained within normal limits. However, the markedly smaller number of Asian participants in that study (n = 22) compared with White women (n = 204) limited the strength of the conclusions.

Over the past two decades, advances in cardiac imaging have led to the development of speckle tracking echocardiography (STE), a technique that enables quantitative assessment of myocardial deformation [16]. Left ventricular global longitudinal strain (LV-GLS) is the principal STE-derived index of systolic function and is more sensitive than LVEF for detecting early myocardial dysfunction [17]. An absolute LV-GLS value below 20% in the presence of preserved LVEF (≥55%) is commonly considered indicative of subclinical myocardial impairment [18]. To date, no studies have specifically evaluated ethnic differ-ences in LV-GLS in healthy pregnant women.

In metropolitan areas such as Milan, where nearly 20% of the population is of foreign origin and approximately 41.7% of the foreign population is of Asian ethnicity [19], pregnancy care increasingly involves women from diverse ethnic backgrounds. This demographic context provides an opportunity to explore ethnicity-related differences in ma-ternal cardiac adaptation. Accordingly, we aimed to assess echocardiographic indices of cardiac morphology and function in two groups of healthy pregnant women—Asians and Caucasians —followed at the Department of Gynaecology and Obstetrics of our institution.

Based on existing evidence from non-pregnant populations, we hypothesized that healthy Asian women in the third trimester of pregnancy may exhibit higher LVEF than European healthy pregnant women. Similarly, given the sensitivity of myocardial deformation indices, we anticipated that LV-GLS might also be higher in Asian compared with European healthy pregnant women.

The primary objective of the present study was to compare conventional and advanced echocardiographic parameters obtained by transthoracic echocardiography, complemented by speckle tracking analysis, in age- and body mass index (BMI)–matched Asian and Caucasian healthy women during the third trimester of pregnancy.

## 2. Materials and Methods

### 2.1. Study Population

This observational, prospective, comparative, monocentric study was conducted at the Obstetrics and Gynaecology Outpatient Clinic of Ospedale San Giuseppe MultiMedica (Milano, Italy) between 15 January 2025 and 1 December 2025. The study enrolled consecutive pregnant women with singleton pregnancy attending routine third-trimester outpatient visits and was designed to compare cardiac structure and function between Asian and Caucasian women matched for age and BMI. The primary study objective was to evaluate whether LVEF and LV-GLS magnitude differ between the two ethnic groups.

For the purpose of the present study, a pregnant woman was considered “healthy” if she had no known pre-existing cardiovascular, respiratory, renal, or metabolic disease; no history of chronic hypertension or diabetes mellitus; no pregnancy-related complications; and normal clinical, laboratory, and echocardiographic findings at the time of enrollment. Eligible participants were women aged between 18 and 45 years, either primiparous or multiparous, with no history of cardiovascular, respiratory, or renal disease and no obstetric complications, including gestational hypertension or gestational diabetes mellitus. European women were included if they were of Caucasian ethnicity, while Asian women were included if they self-identified as Asian. Only women evaluated during the third trimester of pregnancy were considered eligible.

The sampling design consisted of a consecutive, non-random enrollment of eligible participants at a single tertiary-care center. Women meeting the inclusion criteria were invited to participate at the time of their scheduled outpatient visit, and enrollment continued until the predefined sample size for each ethnic group was reached.

Exclusion criteria for both groups included age <18 or >45 years, pregnancy outside the third trimester, pre-existing hypertension or diabetes mellitus, relevant cardiovascular, pulmonary, or renal comorbidities, hemodynamic instability at the time of assessment, inadequate echocardiographic acoustic windows, fetal structural or chromosomal abnormalities, and refusal to participate in the study. Women whose ethnicity did not correspond to the predefined Asian or Caucasian categories were also excluded.

This study was conducted in accordance with the ethical standards of the Declaration of Helsinki and received approval from the local Ethics Committee (reference no. 212/25). All participants provided written informed consent before participation.

### 2.2. Clinical Data Collection

At baseline evaluation, demographic characteristics (age and ethnicity) and anthropometric parameters, including body surface area (BSA), BMI, and modified Haller index (MHI) [20] were recorded. Obstetric data included parity and gestational age at the time of evaluation. Clinical assessment comprised measurement of heart rate, systolic blood pressure, and diastolic blood pressure.

Laboratory investigations included a complete blood count to determine hemoglobin concentration, red cell distribution width (RDW), and the neutrophil-to-lymphocyte ratio (NLR); serum creatinine for estimation of the glomerular filtration rate (eGFR) [21]; and serum glucose levels.

At the same visit, all participants underwent a single blood pressure measurement, a standard 12-lead electrocardiogram (ECG), and a comprehensive transthoracic echocardiographic examination. The echocardiographic protocol included conventional two-dimensional and Doppler assessment, complemented by an extensive speckle-tracking echocardiographic analysis of myocardial deformation properties involving all cardiac chambers, namely both ventricles and both atria. This analysis encompassed left and right ventricular longitudinal deformation as well as left and right atrial strain parameters. Immediately after speckle-tracking echocardiography, a carotid ultrasound examination was also performed. All echocardiographic and vascular ultrasound assessments were conducted on the same day by the same experienced cardiologist.

### 2.3. Conventional Echocardiographic Examination

All echocardiographic examinations were performed using a commercially available Philips Sparq ultrasound system (Philips, Andover, MA, USA) equipped with a 2.5-MHz transducer. Image acquisition was carried out with participants in the left lateral decubitus position. Measurements were obtained in accordance with the recommendations of the American Society of Echocardiography and the European Association of Cardiovascular Imaging [22,23].

Two-dimensional echocardiographic parameters included aortic root and ascending aorta diameters measured using the leading-edge–to–leading-edge convention; relative wall thickness (RWT), calculated as 2 × posterior wall thickness divided by LVEDD; LVMi, calculated using the Devereux formula; left ventricular end-diastolic and end-systolic volume indices (LVEDVi and LVESVi); and LVEF, estimated using the biplane modified Simpson method as an index of LV systolic function [22]. Additional measurements included left atrial volume index (LAVi), right ventricular inflow tract (RVIT), tricuspid annular plane systolic excursion (TAPSE) as an index of right ventricular systolic function, and inferior vena cava (IVC) diameter during spontaneous respiration.

Doppler-derived parameters included the transmitral E/A ratio and the E/average e′ ratio as indices of left ventricular diastolic function and left ventricular filling pressure [23], respectively. Systolic pulmonary artery pressure (sPAP) was estimated using the modified Bernoulli equation: sPAP = 4 × (tricuspid regurgitation velocity)^2^ + estimated right atrial pressure [24]. The severity of concomitant valvular heart disease was graded according to the American Heart Association (AHA) and American College of Cardiology (ACC) recommendations for the management of valvular heart disease [25].

### 2.4. Hemodynamic Assessment

Hemodynamic variables included systolic blood pressure (SBP), diastolic blood pressure (DBP), and mean arterial pressure (MAP), calculated as MAP = DBP + [(SBP − DBP)/3] [26].

Stroke volume (SV) was derived from the product of left ventricular outflow tract (LVOT) area and LVOT time–velocity integral obtained by pulsed-wave Doppler echocardiography. Cardiac output (CO) was calculated as SV multiplied by heart rate [27]. Total peripheral resistance (TPR), expressed in dyne·s/cm^5^, was calculated using the formula TPR = MAP (kPa)/CO (L/min) × 80 [28].

Left ventricular–arterial coupling was assessed by estimating end-systolic pressure (ESP) as 0.9 × brachial systolic blood pressure [29]. Effective arterial elastance index (EaI) was calculated as ESP divided by stroke volume index, and end-systolic elastance index (EesI) was estimated as ESP divided by (LVESVi − V_0_), assuming that V_0_ is negligible compared with LVESVi [30]. Ventricular–arterial coupling was expressed as the EaI/EesI ratio.

### 2.5. Speckle Tracking Echocardiography

Following completion of conventional two-dimensional transthoracic echocardiography, myocardial deformation analysis was performed using high-quality two-dimensional images. Speckle-tracking analysis was conducted sequentially on apical four-chamber, two-chamber, and three-chamber views to assess LV longitudinal strain, and on basal, mid-ventricular, and apical short-axis views to evaluate LV circumferential strain. Image processing was performed using automated function imaging with the Q-Analysis module (Philips QLAB 3.1 software).

According to the QLAB analysis protocol, the LV myocardium was automatically segmented into seven regions for each apical view. Peak systolic strain was defined as the point of maximal myocardial shortening for longitudinal and circumferential strain. LV-GLS and left ventricular global circumferential strain (LV-GCS) were calculated by averaging segmental peak values and displayed in a standardized bull’s-eye plot. Early peak diastolic strain rate was derived from both longitudinal and circumferential strain curves [31].

Right ventricular global longitudinal strain (RV-GLS) was obtained by averaging segmental strain values derived from the apical four-chamber view. Right ventricular free wall longitudinal strain (RV-FWLS) was calculated as the mean strain of the basal, mid, and apical segments of the RV lateral wall, excluding interventricular septal segments, in accordance with current recommendations [32].

Left atrial strain analysis was performed using a biplane approach. The LA endocardial border was automatically divided into seven segments by the QLAB 3.1 software in both the apical four-chamber and two-chamber views. The following parameters were measured: left atrial conduit strain (LAScd), corresponding to the conduit phase; left atrial contractile strain (LASct), corresponding to atrial systole; and left atrial reservoir strain (LASr), defined as the sum of LAScd and LASct. Mean LAScd, LASct, and LASr values were calculated by averaging measurements obtained from the two apical views [33]. From atrial strain curves, strain rate analysis was performed to quantify atrial deformation during the three functional phases of the atrial cycle: peak positive strain rate during ventricular systole, early diastolic strain rate, and late diastolic strain rate. An echocardiographic index of left atrial stiffness was calculated as the ratio between LASr and E/e′ [34].

Right atrial reservoir strain (RASr) was assessed by placing reference points at the lateral and septal sides of the tricuspid annulus and along the endocardial border of the superior right atrial wall, following standardized methodology [35].

Finally, the duration required to complete each speckle-tracking echocardiographic examination was recorded and expressed in minutes.

According to published reference values, normality corresponded to absolute values greater than 20% for LV-GLS [18], 23.6% for LV-GCS [36], 20% for RV-GLS [37], 39% for LASr [38], and 44% for RASr [39].

### 2.6. Carotid Ultrasonography

All participants underwent bilateral carotid ultrasonographic examination using a Philips Sparq ultrasound system (Philips Healthcare, Andover, MA, USA) equipped with a high-frequency 12-MHz linear-array transducer. Carotid imaging was performed in accordance with a standardized and validated scanning protocol [40].

Examinations were conducted with participants in the supine position, with mild neck extension and rotation of the head away from the side being examined to optimize acoustic windows. Longitudinal images of the carotid arteries were acquired at end-diastole, defined by the R wave on the simultaneously recorded electrocardiogram.

Two-dimensional measurements were obtained manually for the following parameters: mean intima–media thickness (IMT) of the left and right common carotid arteries (CCAs) and mean end-diastolic internal diameter (EDD) of the left and right CCAs. All measurements were performed in the distal segment of the CCA, approximately 1 cm proximal to the carotid bifurcation.

Carotid relative wall thickness was calculated as 2 × mean IMT divided by mean CCA-EDD. In addition, the cross-sectional area (CSA) of the CCAs, expressed in mm^2^, was calculated using the following formula: CSA = π × [(2 × mean IMT + mean CCA-EDD)/2]^2^ − π × (mean CCA-EDD/2)^2^, and was used as a surrogate marker of carotid arterial mass [41].

Given that normal IMT values and reference ranges are dependent on age and sex, and that IMT shows a progressive increase with advancing age across all carotid segments [42,43], an age-adjusted threshold was applied. Accordingly, a CCA-IMT value < 0.59 mm was used to define normal intima–media thickness of the common carotid artery in individuals aged 30–39 years [44].

### 2.7. Statistical Analysis

Sample size estimation was performed a priori. A total sample of 40 Asian and 40 Caucasian healthy pregnant women in the third trimester, matched for age and BMI, was calculated to provide 80% statistical power to detect an absolute between-group difference of 4 percentage points in LVEF (i.e., 60% in Caucasian women versus 64% in Asian women) at baseline evaluation [15]. The calculation assumed a standard deviation of 5% for LVEF in both groups, a two-sided *t*-test with equal variances, and a significance level (α) of 0.05.

The distribution of continuous variables was assessed using the Kolmogorov–Smirnov test. Variables with a normal distribution were expressed as mean ± standard deviation, whereas non-normally distributed variables were summarized as median with interquartile range (first and third quartiles). Between-group comparisons were performed using the independent two-tailed Student’s *t*-test for normally distributed variables and the Mann–Whitney U test for non-normally distributed variables. Categorical variables were compared using the chi-square test, as appropriate. For ease of interpretation and to facilitate comparison across parameters, LV-GLS and LV-GCS values were reported as absolute (positive) values throughout the analysis.

Univariate logistic regression analyses were initially performed to explore the associations between selected clinical, laboratory, and echocardiographic variables and the presence of supranormal LVEF, defined as ≥70% [45], as well as enhanced LV-GLS (>20%) [18], in healthy Asian pregnant women. Variables demonstrating a statistically significant association in univariate analyses were subsequently considered for inclusion in multivariate logistic regression models to identify factors independently associated with supranormal LVEF and enhanced LV-GLS. In accordance with the one-in-ten rule for logistic regression modeling, which recommends limiting the number of covariates to one per ten outcome events to reduce the risk of overfitting, the number of variables entered into the multivariate analyses was deliberately restricted. On this basis, four variables were selected a priori for inclusion in the regression models: age as a key demographic variable, BSA as a representative anthropometric parameter, LVEDD as an echocardiographic index of LV size, and the NLR as a laboratory-derived marker of systemic inflammatory status. The selection of these variables was guided by their clinical relevance and by the need to ensure statistical robustness while avoiding overparameterization of the regression models.

Intra- and inter-observer variability for LV-GLS measurements was assessed in a randomly selected subgroup of 15 Asian pregnant women. LV-GLS was reanalyzed by the same cardiologist who performed the original examinations and by a second independent cardiologist. Both analyses were conducted in a blinded fashion. Measurement reproducibility was quantified using the intraclass correlation coefficient (ICC) with corresponding 95% confidence intervals (CI). An ICC value ≥ 0.70 was considered indicative of acceptable reliability.

All statistical analyses were performed using SPSS software, version 28 (SPSS Inc., Chicago, IL, USA). All tests were two-tailed, and a *p*-value < 0.05 was considered statistically significant.

## 3. Results

### 3.1. Study Population and Baseline Characteristics

A total of 80 healthy pregnant women participated in the study, consisting of 40 Asian and 40 Caucasian participants. The baseline clinical characteristics of the two groups of pregnant women are presented in Table 1.

The two groups were comparable in age, with Caucasians having an average age of 32.0 ± 5.2 years and Asians at 32.4 ± 4.1 years. Significant differences were observed in anthropometric measurements, as Asians had a smaller BSA compared to Caucasians. Differences were also observed in chest wall conformation. Although the lateral–lateral (L–L) thoracic diameter was similar between groups, Asian women exhibited a significantly larger A–P thoracic diameter. As a consequence, the MHI—which represents the ratio between the transverse and anteroposterior chest dimensions [20]—was lower in Asian women compared with Caucasian women. The distribution of parity was similar between the groups, with both having a comparable proportion of primiparous and pluriparous women. The gestational age at the time of echocardiographic assessment was also similar in both groups, with both being assessed at approximately 35 weeks of gestation. Cardiovascular risk factors were more common in the Caucasian group, including a significantly higher prevalence of smoking (15%) and dyslipidemia (15%) compared to 0% in the Asian group. No significant difference was observed in the family history of heart disease between the groups. Noncardiovascular comorbidities, such as hypothyroidism, gastroesophageal reflux disease (GERD), and anxiety disorder, were also evaluated, with anxiety disorder being more prevalent in Caucasians (15%) compared to 0% in Asians. Blood tests in the third trimester revealed similar hemoglobin levels between groups, although RDW was significantly lower in Asians. Additionally, the NLR was significantly lower in Asians, suggesting a reduced inflammatory burden compared with Caucasian women. In contrast, creatinine, estimated glomerular filtration rate (eGFR), and fasting glucose levels did not differ significantly between the two groups.

### 3.2. Conventional Echocardiographic Findings

The conventional echocardiographic parameters for both groups are summarized in Table 2.

The Asian group exhibited smaller left ventricular sizes, including lower LVEDD and LVMi, compared to the Caucasian group. In addition, the RWT was slightly higher in Asians. Despite these differences in size, the LV geometry was normal in both groups, with no cases of concentric or eccentric remodeling or hypertrophy observed.

Regarding LV systolic function, Asians had lower left ventricular end-diastolic and end-systolic volumes, and a higher LVEF compared to Caucasians. No significant differences were observed in left ventricular diastolic function, although the E/average e′ ratio was significantly lower in Asians.

Left atrial size was significantly smaller in Asians, including both the anteroposterior and longitudinal diameters, as well as the left atrial volume and left atrial volume index, which were also lower compared to Caucasians.

In terms of right ventricular function, the right ventricular inflow tract was significantly smaller in Asians, while there was no significant difference in TAPSE between the groups. Pulmonary hemodynamics, as measured by sPAP, were similar in both groups.

Finally, aortic dimensions, including the aortic root, ascending aorta, and aortic arch, were smaller in Asians compared to Caucasians.

### 3.3. Hemodynamic Findings

Hemodynamic parameters, including heart rate, blood pressure, stroke volume, cardiac output, and peripheral resistance, were assessed in both groups and are summarized in Table 3.

No significant differences were observed in heart rate between the two groups, indicating that the cardiac rhythm was similar in both Asian and Caucasian participants. Similarly, there were no significant differences in SBP, DBP, or MAP between the groups, suggesting that overall blood pressure regulation did not differ significantly.

However, significant differences were found in stroke volume, with the Asian group exhibiting a lower stroke volume compared to the Caucasian group. This difference in stroke volume contributed to the observed lower cardiac output in the Asian group, as cardiac output is directly related to stroke volume and heart rate.

In terms of total peripheral resistance, the Asian group exhibited higher values, indicating a potential difference in vascular tone or resistance compared to the Caucasian group.

Lastly, significant differences were observed in ventricular–arterial coupling parameters between groups. Asian women exhibited a higher effective arterial elastance index, consistent with increased arterial load relative to left ventricular output. End-systolic elastance indexed also differed significantly between groups and was primarily influenced by the markedly lower LVESVi observed in Asian women, rather than by differences in end-systolic pressure. Consequently, the EaI/EesI ratio was lower in Asian women, indicating a distinct pattern of ventricular–arterial coupling compared with Caucasian women.

### 3.4. Myocardial Strain Parameters

The myocardial strain parameters for both groups are summarized in Table 4.

Asian women consistently exhibited superior myocardial function across various strain measures compared to Caucasian women. Left ventricular longitudinal strain, assessed from the apical four-chamber, two-chamber, and three-chamber views, was significantly higher in Asians. As a result, LV-GLS was also more pronounced in the Asian group, reflecting more efficient left ventricular performance.

Similarly, LV circumferential strain, measured at basal, mid-ventricular, and apical short-axis views, was higher in Asians at all levels. Global circumferential strain was also significantly greater in the Asian group, demonstrating superior circumferential function compared to Caucasians.

Regarding left atrial mechanics, Asian women exhibited significantly higher contractile strain, indicating enhanced left atrial contractile function compared with Caucasian women. In addition, LASr was significantly higher in Asians, reflecting superior left atrial compliance during ventricular systole. Left atrial stiffness, assessed by the LASr/E/e′ ratio, was lower in Asian women, further supporting a more favorable atrial functional profile.

Right ventricular strain parameters, including RV-GLS and RV-FWLS, were significantly higher in Asians, suggesting better right ventricular mechanics. This trend was consistent across both global and regional strain measurements.

Finally, right atrial mechanics were also superior in the Asian group, with higher reservoir and contractile strain values, suggesting enhanced right atrial compliance and contractile performance.

**Figure 1:**
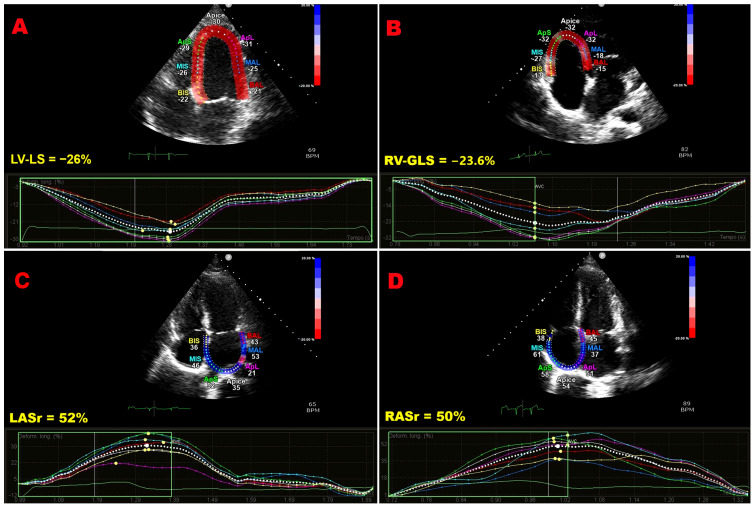
Representative examples of biventricular and biatrial myocardial strain parameters assessed by speckle-tracking echocardiography from the apical 4C-view in an Asian pregnant woman enrolled in the present study. (**A**) LV-LS, left ventricular longitudinal strain. (**B**) RV-GLS, right ventricular global longitudinal strain. (**C**) LASr, left atrial reservoir strain. (**D**) RASr, right atrial reservoir strain. Color-coded segmental strain maps and corresponding strain curves are shown for each chamber.

**Figure 2:**
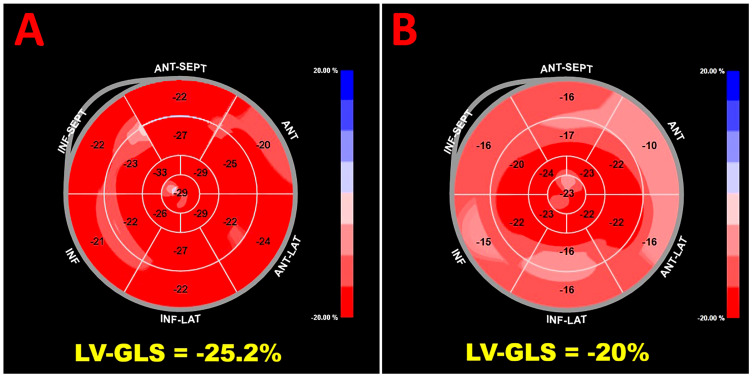
Speckle-tracking echocardiography-derived bull’s-eye plots of LV-GLS obtained in an Asian (**A**) and a Caucasian (**B**) pregnant woman enrolled in the present study. The more intense red color observed in the Asian pregnant woman reflects supranormal longitudinal left ventricular deformation, whereas the lighter red color in the European pregnant woman is mainly attributable to relatively reduced strain in the basal myocardial segments. LV-GLS, left ventricular global longitudinal strain.

Figure 1 shows representative examples of biventricular and biatrial myocardial strain parameters assessed by speckle-tracking echocardiography in an Asian pregnant woman enrolled in the present study, whereas Figure 2 illustrates enhanced and normal LV-GLS values obtained in an Asian and a European pregnant woman, respectively.

### 3.5. Carotid Ultrasound Findings

Significant differences were observed between the Asian and Caucasian groups in several carotid parameters, as detailed in Table 5.

CCA-IMT values detected in both study groups were consistent with normal age-adjusted reference values for the 30–39-year age range [44].

The average common carotid artery end-diastolic diameter was smaller in the Asian group (6.9 ± 0.5 mm) compared to the Caucasian group (7.3 ± 0.4 mm), indicating a smaller arterial diameter in the Asian group.

The carotid intima-media thickness was also significantly lower in Asians [0.46 (0.34–0.68) mm] compared to Caucasians [0.56 (0.35–0.70) mm], suggesting a thinner carotid intima-media layer in the Asian women. Additionally, the carotid relative wall thickness was slightly lower in the Asian group (0.14 ± 0.02) compared to the Caucasian group (0.15 ± 0.02), although this difference was relatively modest.

A marked difference was observed in the carotid CSA, with the Asian group having a significantly smaller CSA (10.7 ± 2.5 mm^2^) compared to the Caucasian group (13.7 ± 2.3 mm^2^), reflecting a smaller overall carotid artery size in Asians.

These results suggest ethnic differences in carotid artery size and structure, with the Asian group showing smaller arterial dimensions and a thinner intima-media layer compared to the Caucasian group.

### 3.6. Univariate and Multivariate Logistic Regression Analyses

The results of the univariate and multivariate logistic regression analyses, evaluating variables associated with supranormal LVEF (≥70%) and enhanced LV-GLS (>20%), are reported in Table 6 and Table 7, respectively.

In univariate analysis, several clinical and echocardiographic parameters showed a significant association with supranormal LVEF (Table 6).

BSA and LVEDD were inversely associated with supranormal LVEF, indicating that lower values of these parameters were more frequently observed in women with LVEF ≥ 70%. Age and NLR were not significantly associated with supranormal LVEF in this initial analysis.

When these variables were entered into the multivariate logistic regression model, LVEDD remained independently associated with supranormal LVEF, whereas the association with BSA was no longer statistically significant.

A second set of regression analyses, including age, BSA, LVEDD, and NLR as covariates, and evaluating their association with enhanced LV-GLS (>20%), is presented in Table 7.

In univariate analysis, lower BSA, lower LVEDD, and lower NLR were significantly associated with enhanced LV-GLS (>20%), whereas age was not associated with the outcome. In the corresponding multivariate model, supranormal LV-GLS (>20%) remained independently associated with lower NLR, while the associations with BSA and LVEDD were no longer statistically significant.

Overall, the regression analyses identified LVEDD and NLR as variables showing independent associations with supranormal LV systolic performance, including enhanced LV-GLS and supranormal LVEF, depending on the covariates included, whereas age was not associated with the outcome in any of the models examined.

### 3.7. Measurement Variability

The intraobserver and interobserver agreement for LV-GLS assessment were excellent, with intraclass correlation coefficients of 0.97 (95% CI, 0.90–0.99) and 0.93 (95% CI, 0.81–0.98), respectively (Supplementary Material S1).

## 4. Discussion

### 4.1. Summary of Findings

In this prospective, age- and BMI–matched cohort of healthy women evaluated during the third trimester of pregnancy, we identified consistent ethnicity-related differences in cardiac structure, hemodynamics, myocardial deformation properties, and carotid vascular characteristics. Compared with Caucasian women, Asian women exhibited smaller cardiac chamber dimensions and lower LV mass, accompanied by reduced ventricular volumes and a higher LVEF. Despite these differences in chamber size, global systolic function assessed by conventional echocardiography remained within the normal range in both groups. Hemodynamic assessment revealed comparable heart rate and blood pressure profiles; however, Asian women demonstrated lower stroke volume and cardiac output in association with higher total peripheral resistance. Importantly, ventricular–arterial coupling, assessed by the EaI/EesI ratio, was significantly lower in Asian women, reflecting a more efficient matching between arterial load and ventricular contractile properties, largely driven by higher end-systolic elastance in the setting of smaller end-systolic volumes.

Beyond conventional echocardiographic parameters, advanced myocardial deformation analysis provided additional insights into ethnic differences in cardiac adaptation to pregnancy. Asian women showed higher LV longitudinal and circumferential strain values, indicating more pronounced myocardial deformation, together with enhanced RV longitudinal mechanics. Atrial mechanics also differed between groups, with Asian women exhibiting higher reservoir strain of both the left and right atria, suggesting differences in atrial functional behavior during pregnancy. These findings highlight that ethnicity-related variations in myocardial performance are detectable not only at the ventricular level but also at the atrial level when assessed using speckle-tracking echocardiography.

In parallel with cardiac findings, vascular ultrasound assessment demonstrated smaller carotid arterial dimensions in Asian women, along with differences in carotid wall characteristics, supporting the presence of ethnic variability in vascular structure during pregnancy. These vascular differences were observed despite similar blood pressure profiles, suggesting that they are unlikely to be driven by hemodynamic confounding alone.

Exploratory regression analyses further demonstrated that indices of cardiac size, particularly LVEDD, and systemic inflammatory status, reflected by NLR, were differentially associated with supranormal LV systolic performance, with LVEDD relating to supranormal LVEF and NLR to enhanced or supranormal LV-GLS (>20%), respectively. The use of multivariate models incorporating age, BSA, ventricular size, and inflammatory markers allowed us to identify independent associations and to reduce the impact of residual confounding.

Taken together, these findings indicate that ethnicity influences multiple aspects of cardiovascular structure and function in healthy pregnancy and underscore the importance of considering ethnic background when interpreting both conventional and advanced cardiovascular imaging parameters in the third trimester.

### 4.2. Comparison with Literature Data

Our findings are broadly consistent with, and extend, previously published data on ethnicity-related differences in cardiac structure and function during pregnancy, most notably those reported by Wilkie et al. [15] in a large retrospective cohort of healthy pregnant women undergoing clinically indicated transthoracic echocardiography. In that study, Asian women exhibited higher LVEF compared with White women, while overall cardiac geometry did not differ significantly across ethnic groups. Importantly, all echocardiographic values remained within conventional normal ranges, leading the authors to conclude that observed differences were unlikely to be clinically significant.

Our results confirm the presence of ethnicity-related differences in conventional echocardiographic indices during pregnancy, particularly higher LVEF and smaller ventricular dimensions in Asian women compared with Caucasian women, despite preserved overall cardiac geometry. From a mechanistic perspective, this combination indicates a systolic phenotype characterized by higher contractile efficiency in the setting of reduced chamber size, rather than a simple volumetric hyperdynamic state. These findings are concordant with data from non-pregnant populations assessed using cardiovascular magnetic resonance (CMR), which demonstrate that South Asian individuals have smaller ventricular volumes, lower myocardial mass, and distinct ventricular remodeling patterns compared with White Europeans, independent of body size and traditional cardiovascular risk factors [46].

However, several methodological and conceptual differences distinguish our study from that of Wilkie et al. First, our investigation was prospective and specifically designed to compare healthy third-trimester Asian and Caucasian pregnant women matched for age and BMI, thereby minimizing confounding related to gestational timing and anthropometric variability. This design allowed us to isolate ethnicity-related physiological mechanisms acting at a comparable stage of maximal cardiovascular load. In contrast, Wilkie et al. included examinations performed across all trimesters and up to 12 weeks postpartum, potentially introducing physiological heterogeneity related to the dynamic cardiovascular adaptations of pregnancy.

Second, and most importantly, our study substantially expands the existing literature by incorporating a comprehensive speckle-tracking echocardiographic assessment of myocardial deformation across all cardiac chambers. Unlike previous studies, which primarily reported global strain indices, we performed a detailed regional analysis of LV myocardial mechanics, including segmental and view-specific assessment of both LV-GLS and LV-GCS. This approach enabled detection of subtle myocardial functional phenotypes that precede or exist independently of changes in ejection fraction. While Wilkie et al. focused exclusively on conventional echocardiographic parameters, our data demonstrate that ethnicity-related differences extend beyond chamber size and LVEF to include left and right ventricular strain, as well as biatrial deformation mechanics. This observation mirrors recent CMR evidence showing higher global longitudinal and circumferential strain in South Asian women despite similar ejection fractions [46], underscoring the ability of strain imaging to uncover subtle functional phenotypes not apparent with volumetric indices alone.

Finally, our study uniquely integrates hemodynamic assessment and carotid ultrasonography, allowing a more holistic evaluation of cardiac–vascular interactions in pregnancy. This multidimensional approach aligns with emerging imaging literature indicating that ethnicity-related cardiovascular differences are not confined to myocardial structure, but also involve ventricular–vascular coupling, loading conditions, and deformation mechanics. Importantly, our findings are consistent with longitudinal hemodynamic data demonstrating that Asian pregnant women exhibit persistently higher peripheral vascular resistance and lower cardiac output compared with White women throughout gestation, despite preserved blood pressure values [47]. Such a high-resistance, lower-volume circulatory adaptation—well documented using non-invasive bioreactance techniques—supports the concept that myocardial performance in Asian women relies more heavily on efficient myocardial shortening and favorable ventricular mechanics rather than volumetric augmentation. These observations reinforce the value of integrating vascular and myocardial assessments to fully characterize ethnicity-specific cardiovascular adaptation during pregnancy.

Together, our findings suggest that ethnicity-related differences in pregnancy-associated cardiovascular adaptation are multidimensional and may not be fully characterized by conventional echocardiographic parameters alone, underscoring the added value of advanced imaging techniques in this setting.

### 4.3. Determinants of Hyperdynamic Systolic Performance and Augmented LV-GLS in Asian Healthy Pregnant Women

The consistently higher LVEF and more pronounced LV-GLS observed in Asian healthy pregnant women likely reflect the convergence of structural, anthropometric, hemodynamic, and biomechanical determinants that collectively favor enhanced systolic efficiency during pregnancy. Rather than indicating a pathological hyperdynamic state, this phenotype appears to represent a physiologically optimized systolic response to pregnancy-related loading conditions. A central contributor is the smaller LV cavity size, evidenced by lower LV end-diastolic diameter and volume, a well-established ethnic characteristic that persists during gestation. From a biomechanical standpoint, ventricular size critically modulates wall stress according to Laplace’s law, whereby wall stress is directly proportional to intracavitary pressure and chamber radius and inversely proportional to wall thickness [48]. In smaller ventricles, a reduced radius results in lower end-systolic wall stress for a given arterial pressure, creating a mechanical environment that facilitates more efficient myocardial fiber shortening.

This favorable geometry is particularly relevant for longitudinally oriented subendocardial fibers, which are highly sensitive to changes in wall stress and loading conditions. Lower wall stress preserves longitudinal deformation, thereby supporting augmented LV-GLS despite the substantial hemodynamic demands of late pregnancy. In this context, LV-GLS may better reflect intrinsic myocardial efficiency than LVEF, which is strongly influenced by chamber size. Even modest pregnancy-related increases in preload can therefore generate disproportionately greater fiber shortening in smaller chambers operating on a steeper portion of the pressure–volume relationship, contributing to enhanced strain values.

Hemodynamic characteristics may also contribute to this pattern. Asian women demonstrated lower stroke volume and cardiac output in the presence of higher peripheral resistance, suggesting a systolic adaptation that relies more on myocardial shortening efficiency than on volumetric output. This pattern is consistent with a ventricular–arterial coupling profile characterized by higher end-systolic elastance relative to arterial elastance, favoring efficient energy transfer. Subtle ethnic differences in autonomic balance and vascular properties, described in non-pregnant populations [49], may persist during pregnancy and modulate ventricular loading conditions in a way that favors longitudinal mechanics.

Beyond structural and hemodynamic factors, biological determinants may play an important role. The lower inflammatory burden observed in Asian women, reflected by lower neutrophil-to-lymphocyte ratios in multivariate models, may contribute to enhanced myocardial deformation. Chronic low-grade inflammation has been linked to subclinical myocardial dysfunction and impaired strain parameters in various clinical settings, even in the absence of overt cardiovascular disease [50,51,52]. Differences in dietary patterns—such as higher intake of anti-inflammatory nutrients—and a lower lifetime burden of atherosclerotic or fibrotic myocardial changes may therefore support more preserved myocardial mechanics in Asian women during pregnancy [53].

Finally, thoracoabdominal configuration may represent a key determinant of the ethnic differences in myocardial deformation observed in the present study. During pregnancy, progressive uterine enlargement displaces the diaphragm cranially, inducing characteristic changes in thoracic geometry. Both the anteroposterior and transverse diameters of the chest wall increase by approximately 2 cm, resulting in a 5–7 cm expansion of the lower rib cage circumference [54]. These anatomical adaptations are most evident in the third trimester, when the heart is displaced upward and leftward with a mild rotation along its longitudinal axis, leading to lateral displacement of the cardiac apex [55]. Importantly, data from our cohort demonstrate that Asian pregnant women exhibited a significantly lower modified Haller index compared with Western women, consistent with a less concave chest wall conformation. In a previous investigation from our study group [56], increased chest wall concavity (higher MHI) was associated with a reversible reduction in left and right ventricular as well as atrial strain parameters during late gestation, despite preserved ejection fraction and normal loading conditions. These findings support the concept that apparent impairment in myocardial deformation during pregnancy may, at least in part, reflect extrinsic mechanical constraints imposed by thoracic geometry rather than intrinsic myocardial dysfunction. Accordingly, it is plausible that the less concave chest wall configuration detected in Asian women in the present study resulted in reduced mechanical restriction of cardiac excursion during late pregnancy. A more favorable thoracoabdominal geometry, characterized by lower diaphragmatic elevation and greater anteroposterior chest dimensions, may allow more complete longitudinal fiber shortening and enhanced myocardial deformation. This mechanism provides a coherent structural explanation for the higher biventricular and biatrial strain values observed in Asian women compared with Western cohorts, despite lower stroke volume and higher vascular resistance. Collectively, these observations highlight thoracic geometry as a critical, yet often overlooked, modulator of cardiac mechanics in pregnancy and underscore the need to interpret strain-derived indices within an ethnicity- and morphology-aware framework.

Collectively, these interrelated anatomical, mechanical, hemodynamic, and biological factors provide a coherent pathophysiological framework explaining why Asian healthy pregnant women exhibit a reproducible pattern of hypercontractile systolic function and enhanced LV-GLS during the third trimester.

### 4.4. Pathophysiological Determinants of Carotid Artery Remodeling in Western Pregnant Women

Carotid artery remodeling observed in Western pregnant women likely reflects the integrated effects of pregnancy-related hemodynamic loading, systemic inflammatory activation, and anthropometric characteristics that modulate vascular adaptation. Rather than representing early vascular pathology, these changes appear to reflect an adaptive remodeling process in response to increased flow and wall stress. Pregnancy is characterized by a substantial increase in circulating blood volume and cardiac output, which exposes the arterial wall to sustained changes in shear stress and transmural pressure. In Western women, who in the present study exhibited larger BSA, higher SV, and lower TPR compared with Asian counterparts, these hemodynamic conditions may favor outward arterial remodeling, as reflected by larger CCA diameter and cross-sectional area.

Beyond purely mechanical factors, low-grade systemic inflammation appears to play a pivotal role in carotid wall remodeling during normal pregnancy. In a previous study conducted by our research group [57], the NLR was identified as the strongest independent determinant of CCA intima–media thickening in healthy pregnant women, exceeding the predictive value of traditional demographic, metabolic, and echocardiographic factors. These findings support the concept that pregnancy represents a state of chronic, mild inflammatory activation capable of inducing measurable vascular wall changes even in the absence of overt cardiovascular disease. In the present study, Caucasian women exhibited higher NLR values, along with larger CCA diameters, increased intima–media thickness, greater relative wall thickness, and larger carotid cross-sectional area compared with Asian women. This pattern suggests that carotid remodeling in Caucasian pregnant women may be more strongly influenced by inflammation-associated wall thickening and outward remodeling, whereas the lower inflammatory burden observed in Asian women may contribute to a more limited and potentially more adaptive vascular remodeling response.

Additionally, anthropometric factors such as higher body surface area and chest wall configuration may indirectly influence carotid dimensions by modulating cardiac output requirements and arterial load. The association between larger LV volumes, higher SV, and increased carotid CSA suggests a coordinated heart–vessel adaptation aimed at maintaining efficient maternal–fetal perfusion.

Overall, carotid artery remodeling in Western pregnant women seems to arise from the interplay between pregnancy-induced hemodynamic stress and inflammation-driven vascular plasticity, with the balance between these mechanisms favoring adaptive arterial enlargement rather than pathological wall thickening. This mechanistic interpretation reinforces the importance of integrating inflammatory biomarkers, vascular geometry, and cardiac function when evaluating cardiovascular adaptation during pregnancy.

### 4.5. Implications for Clinical Practice

The present findings have several relevant implications for the clinical interpretation of echocardiographic examinations during pregnancy. First, the demonstration of consistent ethnicity-related differences in cardiac chamber size, conventional systolic indices, and myocardial deformation parameters highlights the importance of contextualizing echocardiographic measurements according to ethnic background. Asian healthy pregnant women exhibited smaller ventricular dimensions together with higher LVEF and more pronounced LV-GLS, indicating that the application of uniform reference thresholds across ethnically diverse populations may lead to inappropriate classification of cardiac function. In this context, the clear ethnic differences in ventricular size, LVEF, and LV-GLS underscore the need to consider population-specific normative ranges when evaluating cardiac function during late gestation [58]. Applying uniform thresholds across diverse populations risks misclassification, particularly given that Asian women naturally demonstrate greater GLS and higher LVEF compared with Western women. Clinicians should therefore interpret borderline GLS or LVEF values in the context of ethnic background to avoid overestimating subclinical systolic dysfunction.

Second, our results emphasize the relevance of structural and loading conditions in shaping echocardiographic findings during late gestation. Differences in ventricular size, wall stress, and thoracoabdominal configuration may substantially influence myocardial deformation measurements. In women with larger cardiac chambers and greater diaphragmatic elevation—features more frequently observed in Western populations—modest reductions in GLS may reflect transient mechanical or geometric constraints rather than intrinsic myocardial impairment. Conversely, similar GLS values in Asian women, whose baseline myocardial deformation is typically more pronounced, may warrant closer clinical attention. In this context, the incorporation of simple structural indices, such as the modified Haller index, may help clinicians better interpret strain data by accounting for individual thoracic geometry.

In addition, the growing clinical adoption of speckle-tracking echocardiography in obstetric cardiology reinforces the need to establish updated, ethnicity-adjusted reference ranges for both conventional systolic indices and strain-derived parameters. An interpretative framework that integrates ethnic background, cardiac morphology, and hemodynamic state may enhance diagnostic accuracy and improve risk stratification for pregnancy-associated cardiovascular conditions, including hypertensive disorders of pregnancy and peripartum cardiomyopathy.

Finally, our experience demonstrates that speckle-tracking echocardiography is feasible in routine clinical practice. Contrary to earlier concerns regarding technical complexity, operator dependency, and prolonged analysis time [59], our standardized protocol allowed comprehensive strain evaluation—including ventricular and atrial mechanics—to be completed with less than 10 min of additional acquisition and post-processing. This finding indicates that, when performed by experienced operators using optimized workflows, STE can be efficiently incorporated into routine pregnancy echocardiography without significantly extending examination duration, even in high-volume clinical environments.

Overall, these results support a personalized, physiology-based approach to echocardiographic assessment in pregnancy, moving beyond uniform interpretative thresholds toward individualized cardiovascular evaluation.

### 4.6. Study Limitations

Several limitations of the present study should be acknowledged. First, the relatively limited sample size, although based on an a priori power calculation, may restrict the generalizability of the findings and reduce the ability to detect smaller effect sizes for some parameters. Second, the monocentric design may introduce selection bias related to local demographic, clinical, or referral characteristics, thereby limiting external validity. Importantly, the Asian and Caucasian cohorts included in this study cannot be considered representative of the respective ethnic populations at a population level. Participants were recruited from a single metropolitan area and reflect a specific clinical and sociodemographic context; therefore, the observed differences should be interpreted as sample-specific associations rather than definitive ethnic characteristics. Third, the cross-sectional nature of the study and the absence of longitudinal follow-up preclude assessment of temporal changes in cardiac structure and function across pregnancy and prevent evaluation of the persistence or clinical relevance of the observed ethnic differences beyond the third trimester. Accordingly, our findings should be regarded as exploratory and hypothesis-generating, and confirmation in larger, multicenter, population-based cohorts is warranted.

In addition, although a comprehensive echocardiographic protocol was applied, including advanced speckle-tracking analysis, several limitations intrinsic to speckle-tracking echocardiography must be considered. Myocardial strain measurements are influenced by image quality, frame rate selection [60] and operator expertise [61], and remain subject to inter-vendor and software-related variability, which may affect absolute strain values [62]. Furthermore, strain parameters are load-dependent and may be modulated by physiological variations in blood pressure and intravascular volume, even in healthy pregnancy [63]. Finally, extrinsic factors such as thoracic anatomy, breast tissue configuration, and acoustic window quality may impact tracking accuracy, particularly in late gestation [64]. These technical considerations should be taken into account when interpreting strain-derived indices and when comparing results across different populations and studies.

## 5. Conclusions

In healthy women evaluated during the third trimester of pregnancy, ethnicity was associated with consistent differences in cardiac structure, systolic performance, myocardial deformation, hemodynamics, and carotid vascular indices. Compared with Caucasian women, Asian women exhibited smaller left ventricular and atrial dimensions and lower ventricular volumes, accompanied by higher LVEF and more pronounced LV-GLS, as well as enhanced right ventricular longitudinal mechanics and higher reservoir strain of both atria. Despite similar heart rate and blood pressure, Asian women demonstrated lower stroke volume and cardiac output, together with higher total peripheral resistance. Importantly, ventricular–arterial coupling—assessed by the EaI/EesI ratio—was significantly lower in Asian women, indicating a more efficient matching between arterial load and ventricular systolic properties in the setting of smaller ventricular size. Carotid ultrasonography also demonstrated smaller arterial dimensions and differences in wall characteristics between groups. These findings suggest that the interpretation of both conventional echocardiographic parameters and speckle-tracking indices in pregnancy should take ethnic background into account. Larger multicenter studies with longitudinal follow-up are warranted to define ethnicity-specific reference ranges and clarify clinical implications.

## Figures and Tables

**Table 1:** Baseline demographic, anthropometric, obstetrical and clinical characteristics of healthy third-trimester pregnant women, comparing Asian and Caucasian groups.

	Asian Pregnant Women (n = 40)	Caucasian Pregnant Women (n = 40)	*p*-Value
**Demographics, anthropometrics and obstetrics**			
Third-trimester age (yrs)	32.0 ± 5.2	32.4 ± 4.1	0.70
Third-trimester BSA (m^2^)	1.65 ± 0.12	1.77 ± 0.15	**<0.001**
Third trimester BMI (Kg/m^2^)	26.3 (20.8–35.6)	26.6 ± 3.8	0.72
L-L thoracic diameter (cm)	29.8 ± 1.7	30.1 ± 1.8	0.44
A-P thoracic diameter (cm)	13.9 ± 1.4	13.1 ± 1.6	**0.02**
MHI	2.1 ± 0.4	2.3 ± 0.4	**0.03**
Primiparous (%)	21 (52.5)	19 (47.5)	0.82
Pluriparous (%)	19 (47.5)	21 (52.5)	0.82
Gestational week at echocardiographic assessment	35 (28–37)	35.5 (28–38)	0.37
**Cardiovascular risk factors**			
Smoking (%)	0 (0.0)	6 (15.0)	**0.02**
Dyslipidemia (%)	0 (0.0)	6 (15.0)	**0.02**
Family history of heart disease (%)	5 (12.5)	10 (25.0)	0.25
**Noncardiovascular comorbidities**			
Hypothyroidism (%)	7 (17.5)	5 (12.5)	0.75
GERD (%)	1 (2.5)	4 (10.0)	0.36
Anxiety disorder (%)	0 (0.0)	6 (15.0)	**0.02**
**Third trimester blood tests**			
Hemoglobin (g/dL)	11.7 ± 1.0	11.3 ± 1.4	0.14
RDW (%)	13.8 ± 1.7	15.4 ± 3.1	**0.005**
NLR	2.10 ± 0.55	3.9 ± 1.3	**<0.001**
Creatinine (mg/dL)	0.54 (0.28–0.63)	0.56 ± 0.09	0.27
eGFR (mL/min/m^2^)	127.7 (115–159)	125.4 (98.3–136)	0.23
Fasting glucose (mg/dL)	81.0 ± 6.0	79.0 ± 5.7	0.13

Variables with a normal distribution are presented as mean ± standard deviation, while non-normally distributed variables are reported as median (interquartile range). Categorical data arepresented as frequencies and percentages. Statistically significant *p* values are indicated in bold. A–P, antero-posterior; BMI, Body Mass Index; BSA, Body Surface Area; eGFR, Estimated Glomerular Filtration Rate; GERD, Gastroesophageal Reflux Disease; L-L, latero-lateral; MHI, Modified Haller Index (L–L thoracic diameter/A–P thoracic diameter); NLR, Neutrophil-to-Lymphocyte Ratio; RDW, Red Cell Distribution Width.

**Table 2:** Conventional echocardiographic and Doppler-derived indices in healthy third-trimester pregnant women, comparing Asian and Caucasian groups.

Conventional echoDoppler Indices	Asian Pregnant Women (n = 40)	Caucasian Pregnant Women (n = 40)	*p*-Value
*LV size*			
IVS (mm)	7.8 (6–11.5)	8.0 (6.5–10.2)	0.46
PW (mm)	6.6 (5.5–8.5)	6.5 (5.7–7.6)	0.55
LVEDD (mm)	44.2 ± 3.8	46.0 ± 2.2	**0.01**
RWT	0.30 ± 0.04	0.28 ± 0.03	**0.01**
LVMi (mL/m^2^)	58.6 (37.0–102.1)	64.9 ± 12.3	**0.03**
*LV geometric model*			
Normal (%)	39 (97.5)	40 (100)	1.00
LV concentric remodeling (%)	0 (0.0)	0 (0.0)	1.00
LV concentric hypertrophy (%)	0 (0.0)	0 (0.0)	1.00
LV eccentric remodeling (%)	1 (2.5)	0 (0.0)	1.00
*LV systolic function*			
Biplane LVEDV (mL)	48.8 ± 11.1	76.2 ± 17.3	**<0.001**
Biplane LVEDVi (mL/m^2^)	29.4 ± 6.1	43.0 ± 8.0	**<0.001**
Biplane LVESV (mL)	13.8 (7.7–22)	25.0 ± 5.0	**<0.001**
Biplane LVESVi (mL/m^2^)	8.4 (4.9–15)	14.2 ± 2.4	**<0.001**
Biplane LVEF (%)	71.6 (60–80.4)	66.4 (58–70)	**<0.001**
*LV diastolic function*			
E/A ratio	1.37 (0.80–2.50)	1.30 (0.72–1.70)	0.33
E/average e’ ratio	5.8 ± 1.5	9.8 (5.0–14.0)	**<0.001**
*LA size*			
LA A-P diameter (mm)	34.8 ± 4.1	38.2 ± 3.5	**<0.001**
LA longitudinal diameter (mm)	43.7 ± 4.7	47.2 ± 3.9	**<0.001**
LAV (mL)	39.0 ± 9.9	55.4 ± 12.8	**<0.001**
LAVi (mL/m^2^)	23.5 ± 5.7	31.2 ± 5.9	**<0.001**
*Valvulopathies*			
More than mild MR (%)	2 (5.0)	0 (0.0)	0.49
More than mild AR (%)	0 (0.0)	0 (0.0)	1.00
More than mild TR (%)	0 (0.0)	0 (0.0)	1.00
*RV size and systolic function*			
RVIT (mm)	27.4 ± 3.3	30.1 ± 2.7	**<0.001**
TAPSE (mm)	26.4 ± 3.2	25.9 ± 2.5	0.44
*Pulmonary hemodynamics*			
sPAP (mmHg)	22.4 ± 2.8	22.4 ± 2.7	1.00
*Aortic size*			
Aortic root (mm)	27.5 ± 2.5	31.0 ± 1.8	**<0.001**
Ascending aorta (mm)	26.3 ± 2.7	28.8 ± 2.2	**<0.001**
Aortich arch (mm)	22.8 ± 1.8	24.1 ± 1.4	**<0.001**

Continuous variables with a normal distribution are presented as mean ± standard deviation, whereas non- normally distributed variables are expressed as median (interquartile range). Categorical variables are reported as a number (percentage). Statistically significant *p* values are indicated in bold. A-P, antero-posterior; AR, aortic regurgitation; IVS, Interventricular Septum; LA, Left Atrial; LAV, Left Atrial Volume; LAVi, Left Atrial Volume index; LV, Left Ventricular; LVEDD, Left Ventricular End-Diastolic Diameter; LVEDV, Left Ventricular End-Diastolic Volume; LVEDVi, Left Ventricular End-Diastolic Volume index; LVEF, Left Ventricular Ejection Fraction; LVESV, Left Ventricular End-Systolic Volume; LVESVi, Left Ventricular End-Systolic Volume index; LVMi, Left Ventricular Mass index MR, mitral regurgitation; PW, Posterior Wall; RV, Right Ventricular; RVIT, Right Ventricular Inflow Tract; RWT, Relative Wall Thickness; sPAP, Systolic Pulmonary Artery Pressure; TAPSE, Tricuspid Annular Plane Systolic Excursion; TR, tricuspid regurgitation.

**Table 3:** Hemodynamic and ventricular–arterial coupling parameters in healthy third-trimester pregnant women, comparing Asian and Caucasian groups.

	Asian Pregnant Women (n = 40)	Caucasian Pregnant Women (n = 40)	*p*-Value
**Hemodynamics**			
HR (bpm)	85.6 ± 11.6	88.3 ± 8.8	0.24
SBP (mmHg)	109.4 ± 12.3	112.4 ± 8.0	0.20
DBP (mmHg)	69.2 ± 7.4	68.0 ± 4.8	0.39
MAP (mmHg)	82.6 ± 8.1	82.8 ± 4.5	0.89
SV (mL)	45.5 ± 9.6	68.0 (48.9–110)	**<0.001**
CO (L/min)	3.9 ± 0.9	4.9 ± 0.8	**<0.001**
COi (L/min/m^2^)	2.3 (1.3–4.1)	2.8 ± 0.5	**<0.001**
TPR (dyne.sec/cm^5^)	1797.3 (1074.4–3230.9)	1171.1 ± 211.6	**<0.001**
TPRi (dyne.sec/cm^5^)/m^2^	1092.0 ± 287.0	664.2 ± 114.3	**<0.001**
**VAC parameters**			
ESP (mmHg)	98.5 ± 11.1	101.1 ± 7.2	0.22
SVi (mL/m^2^)	27.6 (17.1–41)	38.5 (30.2–53)	**<0.001**
LVESVi (mL/m^2^)	8.4 (4.9–15)	14.2 ± 2.4	**<0.001**
EaI (mmHg/mL/m^2^)	3.7 ± 0.9	2.7 ± 0.4	**<0.001**
EesI (mmHg/mL/m^2^)	12.7 ± 3.9	7.4 (5.5–12)	**<0.001**
EaI/EesI	0.31 ± 0.09	0.37 ± 0.07	**0.001**

Variables with a normal distribution are presented as mean ± standard deviation, whereas non-normally distributed variables are expressed as median (interquartile range). Statistically significant *p* values are indicated in bold. CO, Cardiac Output; COi, Cardiac Output Index; DBP, Diastolic Blood Pressure; EaI, Arterial Elastance Index; EesI, End-Systolic Elastance Index; ESP, End-Systolic Pressure; HR, Heart Rate; LVESVi, Left Ventricular End-Systolic Volume Index; MAP, Mean Arterial Pressure; SBP, Systolic Blood Pressure; SV, Stroke Volume; SVi, Stroke Volume index; TPR, Total Peripheral Resistance; TPRi, Total Peripheral Resistance index; VAC, Ventricular–Arterial Coupling.

**Table 4:** Biventricular and biatrial myocardial mechanics assessed by speckle-tracking echocardiography in healthy third-trimester pregnant women, comparing Asian and Caucasian groups.

	Asian Pregnant Women (n = 40)	Caucasian Pregnant Women (n = 40)	*p*-Value
**LV mechanics**			
*LV longitudinal strain*			
LS apical 4C view (%)	22.4 ± 2.1	20.8 ± 2.2	**0.001**
LSR apical 4C view (s^−1^)	1.31 (1.00–1.60)	1.21 (0.90–1.52)	**0.003**
LS apical 2C view (%)	21.4 (15.0–26.5)	20.4 (14–25.5)	**0.04**
LSR apical 2C view (s^−1^)	1.25 ± 0.20	1.14 ± 0.25	**0.03**
LS apical 3C view (%)	21.8 (17.2–29.1)	20.2 ± 3.3	**0.03**
LSR apical 3C view (s^−1^)	1.35 (1.10–2.0)	1.22 (0.87–1.87)	**0.01**
GLS (%)	21.9 ± 1.9	20.5 ± 2.0	**0.002**
GL-SR (s^−1^)	1.31 (1.06–1.60)	1.19 (0.91–1.50)	**<0.001**
*LV circumferential strain*			
CS basal short-axis view (%)	22.5 ± 3.3	19.3 ± 3.2	**<0.001**
CSR basal short-axis view (s^−1^)	1.61 (1.10–2.40)	1.36 (0.85–2.15)	**<0.001**
CS mid-ventricular short-axis view (%)	26.5 (19.6–35.8)	23.7 (16.8–33)	**0.007**
CSR mid-ventricular short-axis view (s^−1^)	1.80 ± 0.27	1.51 ± 0.28	**<0.001**
CS apical short-axis view (%)	32.1 (24.4–47.5)	29.3 (21.6–44.7)	**0.03**
CSR apical short-axis view (s^−1^)	2.33 ± 0.39	2.05 ± 0.37	**0.001**
GCS (%)	27.0 (21.3–37.8)	24.2 (21.0–28.7)	**<0.001**
GC-SR (s^−1^)	1.91 (1.47–2.53)	1.64 (1.43–1.95)	**<0.001**
**LA mechanics**			
LAScd (%)	33.9 ± 9.8	35.7 (30.2–40.8)	0.27
LASct (%)	10.1 (1.0–21.0)	4.4 (1.0–11.0)	**<0.001**
LASr (%)	44.0 ± 9.1	40.1 ± 4.4	**0.02**
LA-SRs (s^−1^)	2.38 (1.30–5.0)	2.31 (1.70–3.20)	0.59
LA-SRe (s^−1^)	3.01 ± 0.80	2.88 (2.0–5.0)	0.47
LA-SRl (s^−1^)	2.99 (1.50–5.0)	2.56 (1.50–3.30)	**0.006**
**LA stiffness**			
LASr/E/e’	8.1 (3.7–18.4)	4.6 (2.4–9.0)	**<0.001**
**RV mechanics**			
RV-GLS (%)	22.8 (17.6–33.5)	20.6 (13.6–25.9)	**0.01**
RV-GLSR (s^−1^)	1.55 ± 0.27	1.35 (0.81–2.60)	**0.02**
RV-FWLS (%)	24.2 (18.0–34.7)	21.9 (15.0–26.5)	**0.01**
**RA mechanics**			
RAScd (%)	32.5 ± 8.8	28.8 (16.7–36)	**0.03**
RASct (%)	7.0 (1.0–16)	4.8 (1.0–12)	**0.02**
RASr (%)	39.5 (28.5–64.9)	33.6 ± 7.8	**0.002**
RA-SRs (s^−1^)	2.45 (1.40–5.0)	2.27 (1.40–3.0)	0.23
RA-SRe (s^−1^)	2.42 (1.30–4.0)	2.29 (1.30–3.5)	0.42
RA-SRl (s^−1^)	2.57 (1.50–5.0)	2.17 (1.30–3.20)	**0.01**
**Timing STE (min)**	9.8 (7–15)	9.9 (6–15)	0.78

Variables with a normal distribution are presented as mean ± standard deviation, whereas non-normally distributed variables are expressed as median (interquartile range). Statistically significant *p* values are indicated in bold. 2C, Two-Chamber; 3C, Three-Chamber; 4C, Four-Chamber; CS, Circumferential Strain; CSR, Circumferential Strain Rate; e′, Early Diastolic Mitral Annular Velocity; E/e′, Ratio of Early Mitral Inflow Velocity to Early Diastolic Mitral Annular Velocity; GCS, Global Circumferential Strain; GC-SR, Global Circumferential Strain Rate; GLS, Global Longitudinal Strain; GL-SR, Global Longitudinal Strain Rate; LA, Left Atrial; LAScd, Left Atrial Strain during Conduit Phase; LASct, Left Atrial Strain during Contraction Phase; LASr, Left Atrial Reservoir Strain; LA-SRe, Left Atrial Early Diastolic Strain Rate; LA-SRl, Left Atrial Late Diastolic Strain Rate; LA-SRs, Left Atrial Systolic Strain Rate; LS, Longitudinal Strain; LSR, Longitudinal Strain Rate; LV, Left Ventricular; RA, Right Atrial; RAScd, Right Atrial Strain during Conduit Phase; RASct, Right Atrial Strain during Contraction Phase; RASr, Right Atrial Reservoir Strain; RA-SRe, Right Atrial Early Diastolic Strain Rate; RA-SRl, Right Atrial Late Diastolic Strain Rate; RA-SRs, Right Atrial Systolic Strain Rate; RV, Right Ventricular; RV-FWLS, Right Ventricular Free Wall Longitudinal Strain; RV-GLS, Right Ventricular Global Longitudinal Strain; RV-GLSR, Right Ventricular Global Longitudinal Strain Rate; STE, Speckle-Tracking Echocardiography.

**Table 5:** Carotid ultrasound-derived vascular parameters in healthy third-trimester pregnant women, comparing Asian and Caucasian groups.

Carotid Ultrasound Parameters	Asian Pregnant Women (n = 40)	Caucasian Pregnant Women (n = 40)	*p*-Value
Av. CCA-EDD (mm)	6.9 ± 0.5	7.3 ± 0.4	**<0.001**
Av. CCA-IMT (mm)	0.46 (0.34–0.68)	0.56 (0.35–0.70)	**<0.001**
Av. Carotid RWT	0.14 ± 0.02	0.15 ± 0.02	**0.03**
Av. Carotid CSA (mm^2^)	10.7 ± 2.5	13.7 ± 2.3	**<0.001**

Variables with a normal distribution are presented as mean ± standard deviation, whereas non-normally distributed variables are expressed as median (interquartile range). Statistically significant *p* values are indicated in bold. Av., Average; CCA, Common Carotid Artery; CSA, Cross-Sectional Area; EDD, End-Diastolic Diameter; IMT, Intima–Media Thickness; RWT, Relative Wall Thickness.

**Table 6:** Univariate and multivariate logistic regression analyses identifying independent predictors of supra-normal left ventricular ejection fraction (≥70%) in healthy Asian pregnant women.

	Univariate Logistic Regression Analysis		Multivariate Logistic Regression Analysis	
	OR (95% CI)	*p*-Value	OR (95% CI)	*p*-Value
**Age (yrs)**	0.80 (0.62–1.03)	0.10		
**BSA (m^2^)**	0.40 (0.16–0.99)	**0.04**	0.48 (0.16–1.43)	0.19
**LVEDD (mm)**	0.42 (0.18–0.97)	**0.04**	0.39 (0.16–0.97)	**0.04**
**NLR**	0.63 (0.30–1.32)	0.22		

Results are expressed as odds ratios with 95% confidence intervals. Variables with a *p* value < 0.10 in univariate analysis were entered into the multivariate model. Statistically significant *p* values are shown in bold. BSA, Body Surface Area; CI, Confidence Interval; LVEDD: Left Ventricular End-Diastolic Diameter; LVEF, Left Ventricular Ejection Fraction; NLR: Neutrophil-to-Lymphocyte Ratio; OR, Odds Ratio.

**Table 7:** Univariate and multivariate logistic regression analyses identifying independent predictors of enhanced left ventricular global longitudinal strain (>20%) in healthy Asian pregnant women.

	Univariate Logistic Regression Analysis		Multivariate Logistic Regression Analysis	
	OR (95% CI)	*p*-Value	OR (95% CI)	*p*-Value
**Age (yrs)**	0.98 (0.82–1.16)	0.78		
**BSA (m^2^)**	0.41 (0.20–0.86)	**0.02**	0.79 (0.19–3.31)	0.75
**LVEDD (mm)**	0.73 (0.56–0.94)	**0.02**	0.76 (0.34–1.67)	0.49
**NLR**	0.03 (0.00–0.83)	**0.04**	0.04 (0.00–0.87)	**0.04**

Results are presented as odds ratios with 95% confidence intervals. Variables with a *p* value < 0.10 in univariate analysis were included in the multivariate model. Statistically significant *p* values are indicated in bold. BSA, Body Surface Area; CI, Confidence Interval; GLS, Global Longitudinal Strain; LV, Left Ventricular; LVEDD, Left Ventricular End-Diastolic Diameter; NLR, Neutrophil-to-Lymphocyte Ratio; OR, Odds Ratio.

## Data Availability

Data extracted from included studies will be publicly available on Zenodo (https://zenodo.org (accessed on 15 December 2025)).

## Supplementary Materials

The following supporting information can be downloaded at: https://www.mdpi.com/article/10.3390/jcm15020756/s1, Supplementary Material S1: LV-GLS measurement variability in Asian pregnant women.
