# Supplementary material for: Cardiac and Vascular Adaptation During Pregnancy in Asian and Caucasian Women: Insights from a Prospective Cohort Study
Source: J Clin Med. 2026 Jan 16;15(2):756. doi: 10.3390/jcm15020756 (PMC12841702; doi:10.3390/jcm15020756)
Supplement: Supplementary file 1 [file jcm-15-00756-s001.zip › jcm-4083202-Supplementary Materials S1.pdf]

LV-GLS assessment			
PATIENT LIST	INITIAL MEASUREMENT	REMEASUREMENTS	
		Rater 1	Rater 2
1) A.M.	22.1	22.2	22.5
2) P.G.	21.1	21.3	21.4
3) S.P.	19.6	19.5	19.9
4) G.T.	22.5	23.0	23.5
5) M.V.	19.0	18.8	19.6
6) M.C.	23.0	23.6	23.7
7) C.L.	23.7	23.5	23.8
8) G.R.	22.5	23.0	23.6
9) S.V.	23.3	24.0	24.5
10) A.S.	18.9	18.5	18.5
11) M.P.	23.5	23.7	23.0
12) S.M.	21.8	22.5	23.0
13) P.P.	22.7	23.0	23.6
14) E.G.	25.5	25.0	27.0
15) M.B.	20.7	22.0	23.0
ICC (95%CI)		0.97 (0.90-0.99)	0.93 (0.81-0.98)
